# Operationalized Psychodynamic Diagnostic of Adolescents in Light of the New ICD-11 Model of Personality Functioning: Personality Structure, Psychodynamic Conflicts, and Mental Health Problems

**DOI:** 10.3390/children10071095

**Published:** 2023-06-22

**Authors:** Aslı Akın, Kirstin Goth, Inge Seiffge-Krenke, Alexander Obbarius, Sibylle M. Winter, Lea Sarrar

**Affiliations:** 1 Unit for Psychosomatic Medicine, Institute of Cardiothoracic and Vascular Surgery, Deutsches Herzzentrum der Charité—Medical Heart Center of Charité and German Heart Institute Berlin, 13353 Berlin, Germany; asli.akin@charite.de; 2 Charité—Universitätsmedizin Berlin, 10117 Berlin, Germany; 3 Department of Child and Adolescent Psychiatry, Psychiatric University Clinics Basel, 4002 Basel, Switzerland; kirsten.goth@upk.ch; 4 Department of Child and Adolescent Psychiatry, University Clinics Saarland, 66421 Homburg, Germany; 5 Department of Psychology, University of Mainz, 55246 Mainz, Germany; seiffge-krenke@uni-mainz.de; 6 Department of Psychosomatic Medicine, Charité—Universitätsmedizin Berlin, 12200 Berlin, Germany; alexander.obbarius@charite.de; 7 Department of Child and Adolescent Psychiatry, Psychosomatics and Psychotherapy, Charité—Universitätsmedizin Berlin, 13353 Berlin, Germany; sibylle.winter@charite.de; 8 Department of Psychology, Faculty of Sciences, MSB Medical School Berlin, 14197 Berlin, Germany

**Keywords:** adolescence, mental health, personality functioning, psychodynamic diagnostic, personality structure, psychodynamic conflicts

## Abstract

The International Classification of Diseases and Related Health Problems, 11th Revision introduced a fully dimensional approach to personality disorders which conceptionally converges with the long-standing psychodynamic understanding of psychopathology through underlying intra- and interpersonal impairments. In this study, the diagnostic contributions of the two psychodynamic concepts of personality structure and psychodynamic conflicts were investigated through the comparison of self-report data of 189 adolescents with mental health problems and 321 mentally healthy controls. The study results reveal that adolescents with mental health problems show significantly higher impairments in all four domains of personality structure and significantly higher levels of several psychodynamic conflicts. Further, adolescents with different mental health problems significantly differ regarding the impairments in the personality structure domains and several levels of psychodynamic conflicts. While higher structural impairments are shown in adolescents with eating and anxiety disorders, higher levels of the passive self-worth conflict persist in adolescents with depressive disorders, and higher levels of the passive identity conflict are affecting adolescents with eating disorders. The findings suggest that a standardized diagnostic assessment of personality structure and psychodynamic conflicts in adolescent patients could contribute to a deeper understanding of mental health problems and appropriate treatment planning through the detection of underlying intra- and interpersonal impairments.

## 1. Introduction

According to the current state of research, mental health problems affect up to 31% of adolescents worldwide [1]. Furthermore, the onset of adolescence is associated with an increased risk of developing persisting mental disorders [2,3]. At the same time, there is strong heterogeneity in the course of diseases with changing symptoms, remissions, and new manifestations [4]. Therefore, current symptoms of adolescents can only be understood as a temporary expression of the development—they can remain, disappear, or change, making it difficult to derive individual prognoses. From a clinical point of view, next to the diagnostic assessment of symptomatic manifestations, knowledge about underlying and enduring intra- and interpersonal problems of adolescent patients is, therefore, decisive for deriving appropriate and long-term effective psychotherapeutic interventions [5,6].

Taking into account the need for more precise, time-stable, and prognostically relevant diagnosis, the 11th version of the International Statistical Classification of Diseases and Related Health Problems (ICD-11) [7] recently introduced a shift from a categorical to a fully dimensional understanding of personality disorders and adopted a lifespan perspective. Even if the dimensional, holistic, and developmental detection of impairments in the areas of self and interpersonal functioning appears new to the ICD-11 [7], the profound understanding of psychopathology through continuously distributed, early-developed, and temporally persistent intra- and interpersonal impairments is known as a key aspect of the long-standing psychodynamic approach [8].

In the psychodynamic practice, especially, two concepts are viewed as biographically acquired and persisting predispositions for mental health problems, and have been operationalized within the *Operationalized Psychodynamic Diagnostics in Childhood and Adolescence* (OPD-CA) [9]: personality structure and psychodynamic conflicts.

The personality structure represents basic mental functions that are mainly developed in the earliest experiences of infants and children with their primary attachment figures [9]. The emotional resonance and closeness as well as the opportunities for self-determination and identity formation experienced in the first months of life up to middle childhood enable the maturation of the psyches’ ability to regulate the self and its relationships to internal and external objects, which constitutes the personality structure [10,11]. The OPD-CA [9] distinguishes four overarching domains of personality structure (see Table 1). Previous studies revealed that impairments in personality structure are higher in children and adolescents with externalizing disorders [12] and have a mediating effect on the associations between child maltreatment and the development of psychopathology in the lifespan [13,14]. Furthermore, the concept of personality structure is considered a clinically useful measure for Criterion A (level of personality functioning) in the Alternative Model of Personality Disorders (AMPD) of the Diagnostic and Statistical Manual of Mental Disorders (DSM-5) [15], and the severity of problems in personality functioning in the ICD-11 [7,8,16,17]. 

On the basis of the early-acquired personality structure, children go through various developmental tasks from middle childhood to early adolescence, which can lead to so-called psychodynamic conflicts and represent a further important disorder disposition [18]. A psychodynamic conflict is defined as a non-integrated, temporally persistent, contradictory perspective of experience and action [9]. The OPD-CA [9] differentiates seven basic psychodynamic conflicts (see Table 2). Psychodynamic conflicts are unconscious and, therefore, not directly measurable, but can be derived from the modes of coping with them. The mode of coping with a psychodynamic conflict shows up in the experience and behavioral patterns and can be either active, which means over-compensated and object-related, or passive, which means regressive and self-related [9]. Previous empirical investigations have shown that psychodynamic conflicts occur to a much larger extent in adolescents with mental health problems than in mentally healthy adolescents [19]. In addition, differences between the psychodynamic conflicts of adolescents with internalizing and externalizing disorders [12] and between adolescents in inpatient and outpatient treatment [20] were discovered.

In terms of the already mentioned ongoing need for precise, personalized, and prognostically relevant diagnostics beyond symptomatic manifestations, recently addressed in the new dimensional understanding of personality pathology in the ICD-11 [7], the concepts of personality structure and psychodynamic conflicts have become widely established in the psychiatric and psychotherapeutic practice [21,22]. However, there is a lack of controlled empirical research on personality structure and psychodynamic conflicts concerning different mental health problems in the specific life stage of adolescence as a decisive phase for the early detection and treatment of mental disorders [23,24]. The above-mentioned previous studies have mainly examined either the personality structure or psychodynamic conflicts in selected clinical groups and most of them through interview-based observer ratings, although there is evidence that self-reports of psychodynamic constructs may contribute somewhat different information [25]. Therefore, in the present study, self-report instruments were used to examine both the personality structure and psychodynamic conflicts of adolescents with varying mental health problems and mentally healthy controls.

## 2. Materials and Methods

### 2.1. Sample

The total study sample consisted of *N* = 510 participants. About one third (*n* = 189) were adolescents with mental health problems (*M_age_* = 17.9; *SD* = 1.8; 67% female, 31% male, and 2% diverse sex) and two-thirds (*n* = 321) were mentally healthy controls (*M_age_* = 17.1; *SD* = 2.2; 58% female, 41% male, and 1% diverse sex). The participants were recruited between June 2019 and January 2022 through outpatient clinical facilities and educational institutions in Germany. In order to meet the minimum age requirements for the valid measurement of psychodynamic concepts through self-reports [9], the inclusion criterion for both subsamples was an age of participants between 14 and 21 years. Only adolescents who met the diagnostic criteria for at least one syndrome of a mental disorder according to the Patient Health Questionnaire (PHQ-D) [26] were included in the group of participants with mental health problems. The absence of psychopathological syndromes was a criterion for the inclusion in the control group. Through the syndromic-oriented diagnostic assessment, adolescents with mental health problems could be assigned into five subgroups of depressive (*n* = 65), alcohol use (*n* = 53), somatoform (*n* = 29), anxiety (*n* = 25), and eating (*n* = 17) disorder syndromes. Because some participants had multiple mental health syndromes or at least one comorbid mental health syndrome, participants were allocated to the subgroup based on the syndrome with the greatest severity (detailed criteria for determining the main syndrome are explained in the measures section). 

### 2.2. Measures

The OPD-CA2 Structure Questionnaire (OPD-CA2-SQ) [27] was constructed for the self-assessment of the personality structure according to the OPD-CA [9]: 81 items measure impairments on the four domains of personality structure. The items are rated on a five-point scale from no (0) to yes (4). The overall mean score of all items represents the general level of personality structure (“structural functioning”). Higher scores represent higher rates of impairments and, thus, lower levels of structural functioning. In contrast to the scoring guidelines of the original authors, we used mean scores built from the raw scale scores for our calculations. In concordance with previous psychometric investigations [16], a very high reliability of the overall scale (McDonald’s ω = 0.97) and high-to-very-high reliabilities of the sub-dimensions attachment (McDonald’s ω = 0.87), identity (McDonald’s ω = 0.91), interpersonality (McDonald’s ω = 0.91), and control (McDonald’s ω = 0.90) could be confirmed in the present sample. 

The OPD-CA Conflict Questionnaire (OPD-CA-CQ) [28] was developed according to the conflict axis of the OPD-CA [9] and can be used to identify the active and passive modes of coping with the seven psychodynamic conflicts. The self-report questionnaire consists of 28 items that are rated on a five-point scale from no (0) to yes (4). Initial psychometric investigations indicated inconsistent reliabilities of some subscales [29]. Due to the two-item scale mode of the questionnaire, Spearman–Brown was used as the reliability criterion [30]. Subscales were considered in the data analysis if the reliability coefficients were above 0.50 [31]. Seven subscales were excluded from the analyses due to Spearman–Brown coefficients below 0.50: conflict of closeness versus distance in passive (0.34) and active mode (0.28), conflict of submission versus control in passive (−0.05) and active mode (0.32), conflict of taking care of oneself versus being cared for in passive mode (−0.19), oedipal conflict in passive mode (0.25), and identity conflict in active mode (0.48). The following seven subscales turned out to be sufficiently reliable: conflict of taking care of oneself versus being cared for in active mode (0.51), conflict of self-worth in active (0.56) and passive mode (0.69), guilt conflict in active (0.53) and passive mode (0.74), oedipal conflict in active mode (0.66), and identity conflict in passive mode (0.79).

The PHQ-D [26] is designed to screen most common mental disorders on syndrome levels: 58 items measure 16 diseases in five different categories (somatoform, depressive, anxiety, eating, and alcohol use disorders). In addition to categorical diagnosis, an evaluation of the severity of syndromes can be conducted by calculating the scale sum scores. If more than one syndrome was present in a participant, individual severity scales were compared with each other. The syndrome with the greatest severity was defined as the main syndrome and participants were allocated into subgroups based on the main syndrome. Previous psychometric investigations indicated excellent internal consistencies [32]. In the present sample, acceptable-to-good reliabilities were found for the somatoform syndromes scale (McDonald’s ω = 0.79) and depressive syndromes scale (McDonald’s ω = 0.88). For the other diagnostic scales, the calculation of internal consistencies is considered useless, as these are primarily evaluated categorically and with specified jump rules [32].

### 2.3. Data Analyses

The statistical analyses were carried out using IBM SPSS, version 25. The significance level for all statistical tests was set at α = 0.05. Descriptive statistics were determined for all psychodynamic constructs for each study group.

In the first part of data analysis, adolescents with and without mental health problems were compared. Differences in potentially confounding sociodemographic variables (sex, socioeconomic conditions, and age) between adolescents with and without mental health problems were identified using a chi-square (χ^2^) test and independent-sample *t*-tests. In order to compare impairments in personality structure and levels of psychodynamic conflicts between adolescents with and without mental health problems, separate univariate analyses of covariance (ANCOVA) were conducted. Prerequisite assumptions of ANCOVA were tested and met in all models, except for the normality of residuals. Bias-corrected bootstrapping (*n* = 10,000) was used to deal with this violation. For controlling type 1 error rate in multiple comparisons, all significance values were adjusted by Bonferroni–Holm. 

In the second part of data analysis, adolescents with syndromes of somatoform, depressive, anxiety, eating, and alcohol use disorders were compared. χ^2^ test and one-way analysis of variance (ANOVA) models were conducted to estimate differences in sociodemographic variables (sex, socioeconomic conditions, and age) between the five sub-groups. In order to test if adolescents with different syndromes of mental health disorders differ based on personality structure and psychodynamic conflicts, separate univariate ANCOVA models were conducted, using each of the psychodynamic subscales as a dependent variable. The prerequisite assumptions of ANCOVA were checked, and bias-corrected bootstrapping (*n* = 10,000) was used to deal with the non-normality of residuals in some models. When significant differences across groups were identified, post hoc pairwise comparisons were conducted. All significance values were adjusted by Bonferroni–Holm.

In addition to adjusted *p* values *d*, Cramér’s *V* and η_p_^2^ were interpreted as effect sizes, with *d* of 0.1, Cramér’s *V* of 0.2, and η_p_^2^ of 0.01 interpreted as a weak effect; *d* of 0.5, Cramér’s *V* of 0.3, and η_p_^2^ of 0.06 as a moderate effect; and *d* of 0.8, Cramér’s *V* of 0.5, and η_p_^2^ of 0.14 as a strong effect [33].

## 3. Results

Table 3 shows the sociodemographic characteristics of the study participants. Means and standard deviations regarding personality structure and levels of psychodynamic conflicts can be found in Table 4 and Table 5.

No significant differences were observed between adolescents with and without mental health problems concerning sex or socioeconomic conditions (*p* > 0.05). The mean age of the clinical and control group was significantly different, *t*(472.81) = 4.53, *p* < 0.001, and *d* = 0.40. Univariate ANCOVA models with age as a covariate showed significant differences between adolescents with and without mental health problems on ten of twelve psychodynamic constructs. Adolescents with mental health problems reported significantly higher impairments in the overall and four domains of personality structure, all with adjusted *p* = 0.010 with strong effect sizes, and significantly higher levels in the active taking care of oneself versus being cared for, passive self-worth, active guilt, passive guilt, and passive identity conflicts, all with adjusted *p* = 0.010, except for the passive guilt conflict with *p* = 0.015, with weak-to-moderate effect sizes (see Table 4).

The five clinical subgroups of adolescents with depressive, alcohol use, somatoform, anxiety, and eating disorder syndromes showed no statistically significant differences in the socioeconomic conditions and age (*p* > 0.05), but in the sex ratio, χ^2^(8, *N* = 189) = 19.67, *p* = 0.012, and Cramér’s *V* = 0.23. The results of the ANCOVA models controlling for participants’ sex revealed significant differences between adolescents with different mental health problems regarding all domains of personality structure and five psychodynamic conflicts, all with adjusted *p* = 0.010, except for the active oedipal conflict with *p* = 0.014, with moderate-to-strong effect sizes (see Table 5).

## 4. Discussion

According to the psychodynamic approach, impairments in personality structure and pronounced psychodynamic conflicts are early-developed, etiopathologically relevant intra- and interpersonal key aspects of mental health problems [9]. Within the present study, the impairments in personality structure and levels of psychodynamic conflicts were compared between adolescents with and without mental health problems and between adolescents with different mental health problems.

Starting with the comparison of adolescents with and without mental health problems, the results showed that impairments in personality structure and levels of several psychodynamic conflicts are higher in adolescents with mental health problems. 

Both groups differed significantly and with strong effect sizes in terms of impairments in the overall structural functioning, as well as in the four domains of personality structure. This result is in line with findings from research on adult patients [37] and confirms a key assumption of psychodynamic theory: The structural integration provides essential ego abilities that enable communication and interaction with the constantly developing outer and inner world and is a precondition for mental health [9].

Considering the levels of psychodynamic conflicts, the results of this study are in line with the previous findings of Escher et al. [19] and show that adolescents with mental health problems are significantly more affected by psychodynamic conflicts.

Furthermore, the closer examination of adolescents with mental health problems provided some insights into underlying psychodynamic aspects of somatoform, depressive, anxiety, eating, and alcohol use disorder syndromes in adolescence. 

Among other things, the results showed that adolescents with alcohol use disorder syndromes have significantly fewer structural impairments compared to adolescents with syndromes of eating, anxiety, depressive, and somatoform disorders. This finding is in contrast to the literature on adult patients with substance use disorders, which emphasizes higher impairments in personality structure [38]. The examined age group may explain this contrary finding. Epidemiological studies showed that alcohol consumption affects more than half of all adolescents, but, with the transition to adulthood, pathological alcohol consumption decreases from over 30% to 7–20% [39]. The results of our study indicate, in line with epidemiological data, that alcohol consumption in adolescence seems to be a relevant issue, but does not seem to be immediately associated with limited personality functioning, while the persistence of alcohol-related disorders into adulthood is rather connected with deficits in personality structure as described by Subkowski [38]. 

In turn, the study results revealed comparatively increased impairments in the structural functioning of adolescents with syndromes of eating and anxiety disorders. The increased structural impairments of adolescents with syndromes of eating disorders are in line with previous findings on patients with binge eating disorder [40]. Further, it turned out that adolescents with syndromes of anxiety disorders have the highest impairments in the personality structure domain of attachment, which supports the previously discovered connections between insecure attachment experiences and the formation of anxiety problems [41].

Moreover, some insights into specific psychodynamic conflicts of adolescents with syndromes of eating and depressive disorders are derivable. For example, adolescents with depressive and eating disorder syndromes showed the comparatively highest levels of the active taking care of oneself versus being cared for conflict. This finding can help to understand these syndromes against the background of a repressed desire for care. According to Cierpka et al. [42], typical eating disorder behaviors, such as avoiding food intake and disregarding body-related needs, which trigger increased concerns among caregivers, can serve to fulfill a suppressed need for care. Furthermore, previous studies indicated that patients with eating disorders experience less parental care in their childhood [43]. In the matter of adolescents with depressive syndromes, the increased level of the active taking care of oneself versus being cared for conflict supports the fundamental psychoanalytic understanding of depressive feelings as a result of excessive self-sacrifice and disappointment regarding the lack of satisfaction of one’s own needs [44].

Concerning depressive syndromes, we also found a comparatively high level of the passive self-worth conflict, which confirms the psychodynamic assumption of a narcissistic withdrawal in depressive patients [44]. Taken together, the increased levels of psychodynamic conflicts identified in relation to depressive syndromes are in line with the previous research results of Kaufhold et al. [45], in which the dominance of the taking care of oneself versus being cared for and the self-worth conflict could be found in adult patients with chronic depression.

Lastly, adolescents with syndromes of eating disorders also showed the comparatively highest level of the passive identity conflict as well as the comparatively highest impairments in the structural domain of identity. The outstanding relevance of identity problems is in line with the identity theoretical approach pointing out that, due to the lack of opportunities to develop an own identity and independence, the body can become the only field over which an adolescent can self-determine [46]. 

Taken together, the mentioned impairments in personality structure and different levels of psychodynamic conflicts suggest that knowledge regarding psychodynamic concepts can contribute to a precise and holistic diagnostic assessment of mental health problems in adolescence. 

However, some limitations of the presented study must be considered. Above all, the cross-sectional collection of data hinders conclusions about directional influences. Moreover, only self-reports were included, which may have increased the potential for bias through distorted self-perception [47]. By additionally using observer/therapist ratings, for example, through structured clinical interviews, future full-scale studies could investigate the consistency of different assessment methods. In addition, the inadequate internal consistencies that led to the exclusion of clinically relevant psychodynamic conflicts have to be taken into account when interpreting the results. Further, the participants were mainly female and from high or middle-class socioeconomic backgrounds. Additionally, the unequal subsample distribution of adolescents with mental health problems and the reduction of participants to main syndromes despite existing comorbidities have to be considered. All in all, replications of the presented findings through longitudinal investigations with larger and more heterogeneous samples in terms of socioeconomic status, sex, age, and mental disorders using multi-perspective measurement methods are needed.

## 5. Conclusions

Finally, it should be emphasized that the present findings are highly clinically relevant, especially in light of the paradigm shift in the classification of personality disorders according to the level of personality functioning in the AMPD of the DSM-5 [15] and the ICD-11 [7]. The results suggest that the dimensional assessment of intra- and interpersonal problems is not only relevant for the diagnosis of personality pathology but can also contribute to a more precise understanding of various syndromes of mental health problems in adolescents. Thereby, the additional diagnostic knowledge on syndrome-specific impairments in the domains of personality structure and increased psychodynamic conflicts could provide an important basis for the derivation of profound and, therefore, long-term effective treatment methods. For example, the results of the presented study suggest a special need for self-esteem-stabilizing treatment for adolescents with depressive syndromes and identity-enhancing as well as personality-structure-forming interventions for adolescents with syndromes of eating disorders. To summarize, the findings indicate that a standardized assessment of personality structure and psychodynamic conflicts could help to improve clinical prognosis and implement appropriate treatment efforts for adolescent patients.

## Figures and Tables

**Table 1:** Domains of personality structure according to the OPD-CA [9].

Domain	Definition
Attachment	Ability to bond through an inner working model of differentiated and networked inner and outer objects that provides security.
Identity	Ability to perceive oneself and others in a differentiated and time-stable manner.
Interpersonality	Emotional ability to communicate with the external and internal world and to form and establish appropriate relationships.
Control	Ability to regulate and buffer negative feelings, such as displeasure, anger, depression, or listlessness.

**Table 2:** Psychodynamic conflicts according to the OPD-CA [9].

Conflict	Definition
Conflict of closeness versus distance (C1)	Conflict between the desire for dependency and intense closeness in relationships (passive mode) and the pursuit of pronounced emotional independence and fear of closeness (active mode).
Conflict of submission versus control (C2)	Conflict between the tendency to constantly adapt, subordinate, and submit (passive mode), and the desire to rebel, be dominant, and be defiant-aggressive against obligations (active mode).
Conflict of taking care of oneself versus being cared for (C3)	Conflict between the desire for care, security, and the associated dependency in relationships (passive mode), and the defense of the need for care through self-sufficiency and sacrifice for others (active mode).
Conflict of self-worth (C4)	Conflictual self-esteem experience, which leads to a low self-image and experience of inferiority (passive mode) or to a forced self-confidence and the devaluation of others (active mode).
Guilt conflict (C5)	Conflictual experience of responsibility and guilt, which leads to increased self-reproach and excessive loyalty (passive mode) or to a lack of awareness of guilt and excessive accusation of others (active mode).
Oedipal conflict (C6)	Conflictual experience of one’s own gender role and sexuality, which means that eroticism and sexuality cannot be perceived (passive mode) or are overemphasized and determine all areas of life (active mode).
Identity conflict (C7)	Conflictual experience of identity, which leads to disorientation and perplexity (passive mode) or quickly adopted and changing identifications (active mode).

**Table 3:** Sociodemographic characteristics of the participants.

Characteristics	Adolescents with Mental Health Problems (*n* = 189)	Adolescents without Mental Health Problems (*n* = 321)
Age *M* (*SD*)	17.9 (1.8)	17.1 (2.2)
Sex *n* (%)		
female	126 (67%)	187 (58%)
male	59 (31%)	131 (41%)
diverse	4 (2%)	3 (1%)
Socioeconomic status ^a^ *n* (%)		
high	80 (42%)	132 (41%)
medium	62 (33%)	115 (36%)
low	26 (14%)	48 (15%)
very low	21 (11%)	26 (8%)

*Note*. *N* = 510. ^a^ The socioeconomic status was determined through the participants’ reports on their parents’ occupation, while the parental occupation was coded according to the International Standard Classification of Occupation 1988 (ISCO-88) and scaled by the International Socio-Economic Index (ISEI) [34] by using the GESIS transfer code [35]. The socioeconomic status ranges from 16 to 90. The classification into high (66 to 90), medium (50 to 65), low (35 to 49), and very low (16 to 34) is based on the value ranges of Lohmann et al. [36].

**Table 4:** Differences in personality structure and psychodynamic conflicts between adolescents with and without mental health problems.

	Adolescents with Mental Health Problems (*n* = 189)		Adolescents without Mental Health Problems (*n* = 321)		Comparison between Groups ^a^			
Variable	*M*	*SD*	*M*	*SD*	*F*(1, 507)	η_p_^2^	*M_diff_*	BCa 95% CI of *M_diff_*
Overall PS	1.7	0.7	1.1	0.5	125.26 **	0.20	0.6	[0.49, 0.72]
Attachment	1.6	0.7	1.0	0.5	102.23 **	0.17	0.6	[0.47, 0.71]
Identity	1.7	0.7	1.2	0.6	97.75 **	0.16	0.6	[0.45, 0.70]
Interpersonality	1.7	0.7	1.1	0.5	108.80 **	0.18	0.6	[0.47, 0.70]
Control	1.7	0.8	1.0	0.6	120.02 **	0.19	0.7	[0.57, 0.83]
C3a	1.2	0.9	0.8	0.8	24.26 **	0.05	0.4	[0.22, 0.52]
C4a	1.6	1.2	1.4	1.1	1.06	0.00	0.1	[−0.10, 0.31]
C4p	1.5	1.1	1.0	0.9	38.39 **	0.07	0.6	[0.38, 0.74]
C5a	0.4	0.8	0.2	0.6	16.03 **	0.03	0.3	[0.12, 0.39]
C5p	1.7	1.1	1.5	1.0	6.02 *	0.01	0.2	[0.05, 0.42]
C6a	0.8	0.9	0.7	0.9	2.99	0.01	0.1	[−0.02, 0.31]
C7p	1.4	1.2	0.8	1.0	34.35 **	0.06	0.6	[0.37, 0.78]

*Note. N* = 510. PS = personality structure. C3a = active conflict of taking care of oneself versus being cared for. C4a = active conflict of self-worth. C4p = passive conflict of self-worth. C5a = active guilt conflict. C5p = passive guilt conflict. C6a = active oedipal conflict. C7p = passive identity conflict. *M* = mean. *SD* = standard deviation. *F* = test value. η_p_^2^ = partial eta squared. *M_diff_* = mean difference. BCa 95% CI = bias corrected and accelerated 95% confidence interval. The scales of personality structure and psychodynamic conflicts vary between 0 (no) and 4 (yes), so that higher mean scores represent higher impairments in personality structure and higher levels of psychodynamic conflicts. ^a^ Age was involved as covariate. ** adjusted *p* ≤ 0.010. * adjusted *p* ≤ 0.050.

**Table 5:** Differences in personality structure and psychodynamic conflicts between adolescents with different mental health problems.

	Adolescents with Anxiety Disorder Syndromes (1)	Adolescents with Alcohol Use Disorder Syndromes (2)	Adolescents with Depressive Disorder Syndromes (3)	Adolescents with Eating Disorder Syndromes (4)	Adolescents with Somatoform Disorder Syndromes (5)	Comparison between Groups ^a^		
Variable	*M (SD)*	*M (SD)*	*M (SD)*	*M (SD)*	*M (SD)*	*F*(4, 183)	η_p_^2^	Post hoc tests
Overall PS	2.0 (0.7)	1.2 (0.5)	1.9 (0.6)	2.1 (0.7)	1.7 (0.5)	13.35 **	0.23	1 > 2; 1 > 5; 2 < 3; 2 < 4; 2 < 5; 4 > 5
Attachment	2.1 (0.8)	1.1 (0.5)	1.8 (0.7)	1.9 (0.8)	1.6 (0.5)	12.84 **	0.22	1 > 2; 1 > 5; 2 < 3; 2 < 4; 2 < 5
Identity	2.0 (0.8)	1.3 (0.6)	1.9 (0.7)	2.3 (0.7)	1.7 (0.7)	10.27 **	0.18	1 > 2; 2 < 3; 2 < 4; 2 < 5; 4 > 5
Interpersonality	2.0 (0.7)	1.2 (0.5)	1.9 (0.7)	2.1 (0.7)	1.6 (0.5)	12.73 **	0.22	1 > 2; 1 > 5; 2 < 3; 2 < 4; 2 < 5; 3 > 5; 4 > 5
Control	2.2 (0.8)	1.2 (0.7)	1.8 (0.7)	2.2 (0.8)	1.7 (0.7)	10.96 **	0.19	1 > 2; 1 > 5; 2 < 3; 2 < 4; 2 < 5; 4 > 5
C3a	1.3 (1.2)	0.9 (0.6)	1.4 (0.9)	1.7 (09)	0.8 (0.7)	6.68 **	0.13	1 > 5; 2 < 3; 2 < 4; 3 > 5; 4 > 5
C4a	1.7 (1.4)	1.7 (1.1)	1.3 (1.1)	2.1 (1.1)	1.6 (1.2)	2.02	0.04	/
C4p	1.8 (1.2)	0.8 (0.7)	1.9 (1.1)	1.7 (1.2)	1.3 (0.7)	9.93 **	0.18	1 > 2; 1 > 5; 2 < 3; 2 < 4; 3 > 5
C5a	0.7 (1.2)	0.2 (0.7)	0.5 (0.8)	1.0 (0.9)	0.2 (0.4)	5.03 **	0.10	1 > 5; 2 < 4; 3 < 4; 3 < 5; 4 > 5
C5p	1.6 (1.1)	1.4 (0.9)	1.7 (1.1)	2.2 (1.1)	2.1 (1.1)	2.35	0.05	/
C6a	0.7 (1.0)	1.1 (1.0)	0.6 (0.9)	1.0 (0.9)	0.9 (0.7)	3.21 *	0.07	2 > 3
C7p	1.5 (1.4)	0.8 (0.9)	1.7 (1.2)	2.2 (1.0)	1.3 (1.2)	6.39 **	0.12	1 > 2; 2 < 3; 2 < 4; 4 > 5

*Note*. *N* = 189 (*n* = 25 adolescents with anxiety disorder syndromes; *n* = 53 adolescents with alcohol use disorder syndromes; *n* = 65 adolescents with depressive disorder syndromes; *n* = 17 adolescents with eating disorder syndromes; and *n* = 29 adolescents with somatoform disorder syndromes). PS = personality structure. C3a = active conflict of taking care of oneself versus being cared for. C4a = active conflict of self-worth. C4p = passive conflict of self-worth. C5a = active guilt conflict. C5p = passive guilt conflict. C6a = active oedipal conflict. C7p = passive identity conflict. *M* = mean. *SD* = standard deviation. *F* = test value. η_p_^2^ = partial eta squared. The scales of personality structure and psychodynamic conflicts vary between 0 (no) and 4 (yes), so that higher mean scores represent higher impairments in personality structure and higher levels of psychodynamic conflicts. The numbers in parentheses in group names refer to the numbers used in illustrating statistically significant differences in the post hoc tests. ^a^ Sex was involved as covariate. ** adjusted *p* ≤ 0.010. * adjusted *p* ≤ 0.050.

## Data Availability

The datasets for this manuscript are not publicly available to protect the participants’ confidentiality. Requests to access the datasets should be directed to L.S.

## References

[1] Silva S.A., Silva S.U., Ronca D.B., Gonçalves V.S.S., Dutra E.S., Carvalho K.M.B. (2020). Common mental disorders prevalence in adolescents: A systematic review and meta-analyses. PLoS ONE.

[2] Göbel K., Ortelbach N., Cohrdes C., Baumgarten F., Meyrose A.K., Ravens-Sieberer U., Scheithauer H. (2022). Co-occurrence, stability and manifestation of child and adolescent mental health problems: A latent transition analysis. BMC Psychol..

[3] Gustavson K., Knudsen A.K., Nesvåg R., Knudsen G.P., Vollset S.E., Reichborn-Kjennerud T. (2018). Prevalence and stability of mental disorders among young adults: Findings from a longitudinal study. BMC Psychiatry.

[4] Oldehinkel A.J., Ormel J. (2023). Annual research review: Stability of psychopathology: Lessons learned from longitudinal population surveys. J. Child Psychol. Psychiatry.

[5] Barry C.T., Kim H., Halter B.M., Matson J.L. (2023). Developmental psychopathology. Handbook of Clinical Child Psychology. Integrating Theory and Research into Practice.

[6] Schwetz A., Höfner C., Hochgerner M. (2022). Auf dem Weg zur Psychotherapeutischen Diagnostik: Notwendigkeit und Chance. On the way to psychotherapeutic diagnostics: Necessity and opportunity. Psychotherapeutische Diagnostik.

[7] World Health Organisation (2019). International Classification of Diseases and Related Health Problems.

[8] Blüml V., Doering S. (2021). ICD-11 Personality disorders: A psychodynamic perspective on personality functioning. Front. Psychiatry.

[9] Task Force OPD-CA-2 (2020). OPD-CA-2 Operationalisierte Psychodynamische Diagnostik im Kindes- und Jugendalter: Grundlagen und Manual [OPD-CA-2 Operationalized Psychodynamic Diagnosis in Childhood and Adolescence: Theoretical Basis and User Manual].

[10] Ehrenthal J.C., Benecke C., Kramer U. (2019). Chapter 15—Tailored treatment planning for individuals with personality disorders: The Operationalized Psychodynamic Diagnosis (OPD) approach. Case Formulation for Personality Disorders.

[11] Resch F., Parzer P., Resch F., Parzer P. (2021). Psychic Structure, personality and psychodynamics. Adolescent Risk Behavior and Self-Regulation: A Cybernetic Perspective.

[12] Winter S., Jelen A., Pressel C., Lenz K., Lehmkuhl U. (2011). Klinische und empirische Befunde zur OPD-KJ [Clinical and empirical findings with the OPD-CA]. Prax. Kinderpsychol. Kinderpsychiatr..

[13] Ernst M., Brähler E., Kampling H., Kruse J., Fegert J.M., Plener P.L., Beutel M.E. (2022). Is the end in the beginning? Child maltreatment increases the risk of non-suicidal self-injury and suicide attempts through impaired personality functioning. Child Abus. Negl..

[14] Freier A., Kruse J., Schmalbach B., Zara S., Werner S., Brähler E., Fegert J.M., Kampling H. (2022). The mediation effect of personality functioning between different types of child maltreatment and the development of depression/anxiety symptoms—A German representative study. J. Affect. Disord..

[15] American Psychiatric Association (2013). Diagnostic and Statistical Manual of Mental Disorders.

[16] Schrobildgen C., Goth K., Weissensteiner R., Lazari O., Schmeck K. (2019). Der OPD-KJ2-SF—Ein Instrument zur Erfassung der Achse Struktur der OPD-KJ-2 bei Jugendlichen im Selbsturteil [The OPD-CA2-SQ—An instrument for assessing the structure axis of the OPD-CA-2 in adolescents’ self-assessment]. Z. Kinder- Jugendpsychiatrie Psychother..

[17] Zimmermann J., Müller S., Bach B., Hutsebaut J., Hummelen B., Fischer F. (2020). A common metric for self-reported severity of personality disorder. Psychopathology.

[18] Asbrand J., Schmitz J. (2023). Lehrbuch Klinische Kinder- und Jugendpsychologie und Psychotherapie [Textbook Clinical Child and Adolescent Psychology and Psychotherapy].

[19] Escher F., Sarrar L., Seiffge-Krenke I. (2021). Differences in psychodynamic conflicts between healthy adolescents and adolescent patients: Results with the conflict questionnaire. Nord. Psychol..

[20] Mayer S., Rathgeber M., Sommer T., Winter S., Seiffge-Krenke I. (2021). Diagnostische Arbeit mit dem Befundbogen der Konfliktachse der OPD-KJ: Empirische Ergebnisse an stationären und ambulanten Patient_innen [Diagnostic work with the conflict axis of the OPD-CA: Empirical results on inpatients and outpatients]. Z. Kinder- Jugendpsychiatrie Psychother..

[21] Sammet I., Himmighoffen H., Brücker J., Dreher C., Olshausen Küchenhof C., Wilmers F., Wolf A., Zell P., Küchenhoff J. (2012). OPD in der Klinik: Ein Algorithmus für die Therapieplanung mit der Operationalisierten Psychodynamischen Diagnostik (OPD-2) [OPD in the hospital: An algorithm for structuring the diagnostic process with the Operationalized Psychodynamic Diagnostics OPD-2]. Z. Psychosom. Med. Psychother..

[22] Both L.M., Bastos A.G., Freitas L.H.M. (2019). Operationalized psychodynamic diagnosis: A systematic review of the literature. Trends Psychiatry Psychother..

[23] Kaess M., Cavelti M., Fegert J., Resch F., Plener P., Kaess M., Döpfner M., Konrad K., Legenbauer T. (2021). Frühintervention psychischer Störungen [Early intervention of mental disorders]. Psychiatrie und Psychotherapie des Kindes- und Jugendalters.

[24] Skokauskas N., Fung D., Flaherty L.T., von Klitzing K., Pūras D., Servili C., Dua T., Falissard B., Vostanis P., Moyano M.B. (2019). Shaping the future of child and adolescent psychiatry. Child Adolesc. Psychiatry Ment. Health.

[25] Dinger U., Schauenburg H., Hörz S., Rentrop M., Komo-Lang M., Klinkerfuß M., Köhling J., Grande T., Ehrenthal J.C. (2014). Self-Report and observer ratings of personality functioning: A study of the OPD system. J. Personal. Assess..

[26] Löwe B., Spitzer R.L., Zipfel S., Herzog W. (2002). Gesundheitsfragebogen für Patienten (PHQ-D). Manual und Testunterlagen [Patient Health Questionnaire (PHQ-D). Manual and Test Documents].

[27] Goth K., Schrobildgen C., Schmeck K. (2018). Das Inventar OPD-KJ2-SF (Operationalisierte Psychodynamische Diagnostik im Kindes- und Jugendalter—Strukturfragebogen). Deutschsprachige Version: Ein Fragebogen zur Selbstbeantwortung für die Erfassung der Persönlichkeitsstruktur im Jugendalter—Kurzbeschreibung. [The Inventory OPD-CA2-SQ (Operationalized Psychodynamic Diagnostics in Childhood and Adolescence—Structural Questionnaire). German Version: A Questionnaire for Self-Response for the Assessment of Personality Structure in Adolescence—Short Description]. https://academic-tests.com.

[28] Seiffge-Krenke I., Escher F.J. (2021). Entwicklung eines Fragebogens zur Selbst- und Fremdeinschätzung von OPD-KJ-Konflikten durch Patient_innen und Therapeut_innen [Psychodynamic conflicts: Development of a questionnaire for the assessment by patients and their therapists]. Z. Kinder- Jugendpsychiatrie Psychother..

[29] Cropp C., Claaßen B. (2021). Reliabilität und Validität des OPD-KJ-Konfliktfragebogens bei stationär behandelten Jugendlichen [Reliability and validity of the OPD-CA conflict questionnaire in an adolescent inpatient sample]. Z. Kinder- Jugendpsychiatrie Psychother..

[30] Eisinga R., te Grotenhuis M., Pelzer B. (2013). The reliability of a two-item scale: Pearson, Cronbach or Spearman-Brown?. Int. J. Public Health.

[31] Blanz M. (2021). Forschungsmethoden und Statistik für die Soziale Arbeit: Grundlagen und Anwendungen [Research Methods and Statistics for Social Work: Fundamentals and Applications].

[32] Gräfe K., Zipfel S., Herzog W., Löwe B. (2004). Screening psychischer Störungen mit dem “Gesundheitsfragebogen für Patienten (PHQ-D)“ [Screening for mental disorders using the “Patient Health Questionnaire (PHQ-D)”]. Diagnostica.

[33] Cohen J. (1988). Statistical Power Analysis for the Behavioral Sciences.

[34] Ganzeboom H.B., De Graaf P.M., Treiman D.J. (1992). A standard international socio-economic index of occupational status. Soc. Sci. Res..

[35] Geis A. (2011). Handbuch für die Berufsvercodung [Handbook for Occupational Coding].

[36] Lohmann H., Spieß C.K., Feldhaus C. (2009). *Der Trend zur Privatschule geht an bildungsfernen Eltern vorbei* [The trend towards private schools is bypassing educationally disadvantaged parents]. DIW Wochenber..

[37] Vierl L., Juen F., Benecke C., Hörz-Sagstetter S. (2023). Exploring the associations between psychodynamic constructs and psychopathology: A network approach. Personal. Ment. Health.

[38] Subkowski P., von Heyden M., Jungaberle H., Majić T. (2018). Psychodynamik des Rauschs und der Sucht [Psychodynamics of intoxication and addiction]. Handbuch Psychoaktive Substanzen.

[39] Laucht M., Blomeyer D., Buchmann A., Batra A., Mann K., Singer M.V. (2011). Alkohol und Tabak in der Adoleszenz [Alcohol and tobacco in adolescence]. Alkohol und Tabak: Grundlagen und Folgeerkrankungen.

[40] Van Riel L., Van den Berg E., Polak M., Geerts M., Peen J., Ingenhoven T., Dekker J. (2020). Personality functioning in obesity and binge eating disorder: Combining a psychodynamic and trait perspective. J. Psychiatr. Pract..

[41] Van Assche L., Van de Ven L., Vandenbulcke M., Luyten P. (2020). Ghosts from the past? The association between childhood interpersonal trauma, attachment and anxiety and depression in late life. Aging Ment. Health.

[42] Cierpka M., Reich G., Becker S., Beisel S., Benninghoven D. (2010). Psychotherapie der Essstörungen: Krankheitsmodelle und Therapiepraxis [Psychotherapy of Eating Disorders: Disease Models and Therapy Practice].

[43] Monteleone A.M., Ruzzi V., Patriciello G., Pellegrino F., Cascino G., Castellini G., Steardo L., Monteleone P., Maj M. (2020). Parental bonding, childhood maltreatment and eating disorder psychopathology: An investigation of their interactions. Eat. Weight Disord.—Stud. Anorex. Bulim. Obes..

[44] Leuzinger-Bohleber M., Jiménez J.P., Botto A., Fonagy P. (2021). Contemporary psychodynamic theories on depression. Etiopathogenic Theories and Models in Depression.

[45] Kaufhold J., Negele A., Leuzinger-Bohleber M., Kallenbach L., Ernst M., Bahrke U. (2017). Zur Konfliktdynamik bei chronischer Depression—Ergebnisse zur Konflikt- und Strukturachse der OPD in der LAC-Studie [Conflict dynamics in chronic depression—Results of the conflict and structure axis using the OPD in the LAC study]. Z. Psychosom. Med. Psychother..

[46] Klotter C., Klotter C. (2016). Essstörungen und Identität [Eating disorders and identity]. Identitätsbildung über Essen.

[47] Demetriou C., Ozer B.U., Essau C.A., Cautin R.L., Lilienfeld S.O. (2015). Self-report questionnaires. The Encyclopedia of Clinical Psychology.

